# Online reporting for malaria surveillance using micro-monetary incentives, in urban India 2010-2011

**DOI:** 10.1186/1475-2875-11-43

**Published:** 2012-02-13

**Authors:** Rumi Chunara, Vina Chhaya, Sunetra Bane, Sumiko R Mekaru, Emily H Chan, Clark C Freifeld, John S Brownstein

**Affiliations:** 1 Department of Pediatrics, Harvard Medical School, Boston, Massachusetts, USA; 2 Children's Hospital Informatics Program, Division of Emergency Medicine, Children's Hospital Boston, Massachusetts, USA; 3 ASPH/CDC Allan Rosenfield Global Health Fellowship Program, Washington, DC, USA; 4 Department of Epidemiology, Boston University School of Public Health, Boston, USA; 5 Department of Biomedical Engineering, Boston University College of Engineering, Boston, Massachusetts, USA; 6 Department of Epidemiology, Biostatistics and Occupational Health, McGill University, Montreal, Quebec, Canada

**Keywords:** Self-report, Participatory, Incentives, Internet, Surveillance

## Abstract

**Background:**

The objective of this study was to investigate the use of novel surveillance tools in a malaria endemic region where prevalence information is limited. Specifically, online reporting for participatory epidemiology was used to gather information about malaria spread directly from the public. Individuals in India were incentivized to self-report their recent experience with malaria by micro-monetary payments.

**Methods:**

Self-reports about malaria diagnosis status and related information were solicited online via Amazon's Mechanical Turk. Responders were paid $0.02 to answer survey questions regarding their recent experience with malaria. Timing of the peak volume of weekly self-reported malaria diagnosis in 2010 was compared to other available metrics such as the volume over time of and information about the epidemic from media sources. Distribution of *Plasmodium *species reports were compared with values from the literature. The study was conducted in summer 2010 during a malaria outbreak in Mumbai and expanded to other cities during summer 2011, and prevalence from self-reports in 2010 and 2011 was contrasted.

**Results:**

Distribution of *Plasmodium *species diagnosis through self-report in 2010 revealed 59% for *Plasmodium vivax*, which is comparable to literature reports of the burden of *P. vivax *in India (between 50 and 69%). Self-reported *Plasmodium falciparum *diagnosis was 19% and during the 2010 outbreak and the estimated burden was between 10 and 15%. Prevalence between 2010 and 2011 via self-reports decreased significantly from 36.9% to 19.54% in Mumbai (*p *= 0.001), and official reports also confirmed a prevalence decrease in 2011.

**Conclusions:**

With careful study design, micro-monetary incentives and online reporting are a rapid way to solicit malaria, and potentially other public health information. This methodology provides a cost-effective way of executing a field study that can act as a complement to traditional public health surveillance methods, offering an opportunity to obtain information about malaria activity, temporal progression, demographics affected or *Plasmodium*-specific diagnosis at a finer resolution than official reports can provide. The recent adoption of technologies, such as the Internet supports self-reporting mediums, and self-reporting should continue to be studied as it can foster preventative health behaviours.

## Background

Since the early 1900's, estimates of pre-intervention malaria distribution and risk have utilized a variety of data sources including national records of disease; vector presence and absence; sickle cell incidence; and spleen, parasite, sporozoite, and biting rates [1]. The World Health Organization has typically computed malaria burden using national disease notifications to regional offices. These data sources and passive surveillance methods do not precisely define the population at risk for malaria. Recently, the increased prevalence of asymptomatic malaria infection due to the acquisition of functional immunity [2] has forced epidemiologists to develop surveillance methods designed to understand patterns of mild clinical malaria. Additionally, while effective therapies have been in place for five years in India [3], understanding spread of the disease and issues such as proportion of mortality by age and geographic distribution of plasmodium infection types, are persistent issues that have implications for prevention and treatment strategies. Thus, newer techniques have incorporated epidemiological, geographical and demographic data [4] to provide more robust estimates of malaria impact. Nonetheless, recent studies have shown that underestimation persists; inadequacies in conventional measurement of malaria-associated deaths [5] underscore the constant need for refinement of surveillance methods.

Crowd-sourcing is an emerging concept where the goal is to outsource tasks traditionally performed by one employee to a large disperse and often anonymous group. In, public health, crowd-sourcing provides a new avenue for disease surveillance [6], especially given the recent ubiquity of information technology tools that can automate and accelerate the data collection process. Participants are typically motivated to report public health events by the possibility of targeted and rapid interventions for themselves and their communities [7]. While many crowd-sourcing efforts [8-10] have proved successful without providing direct monetary compensation to their participants, stimulating participation remains a key challenge for many projects. Small-monetary compensation, (even just as effectively as larger amounts) can increase the rate and quality of paper survey responses as well as drug adherence in patients [11,12]. Amazon's Mechanical Turk (AMT) is a market in which anyone can post micro-tasks and the responders ("Turkers") receive a stated fee for each task completed. This paper describes a study using Amazon's Mechanical Turk to investigate the potential of micro-monetary incentives for public health reporting by the general public.

## Methods

In 2010, malaria information from Turkers was solicited from June through August 2010 during a malaria outbreak in Mumbai. In 2011, geographic coverage of the study was expanded to include Mumbai, Delhi, Ahmadabad and Hyderabad, all of which are urban centers affected by Malaria. The Human Intelligence Task (HIT) survey used in 2010 had seven questions (Table 1), and in 2011 this was refined, keeping the key questions and adding questions regarding specific diagnosis date, malaria status of household members and malaria awareness (Additional File 1). In 2010, HITs were posted at intervals of approximately two weeks, gathering data from July 15 to August 26 in batches to audiences limited to Mumbai (six batches). In 2011, the surveys were posted in a single batch to avoid issues of repeat users. Mumbai and Hyderabad surveys were available to Turkers from June 20 to August 26, and Ahmadabad and Delhi from July 14 to August 26. More details of this change are described in Additional File 2. The Amazon interface allows for the ability to restrict responders by country, and we restricted to a specific city by requesting in the survey description that users only respond if they lived in that city. Turkers were paid $0.02 to answer the survey and total cost for the studies in this paper were $20.75 (2010) and $16.98 (2011), including Amazon's fees. In order to assess the accessibility and accuracy of this informal and self-reported public health information, the temporal course of responses, proportion of reports of each *Plasmodium *type, and prevalence between years was examined. Because official malaria case counts were unavailable at time of writing and in general not released at the temporal resolution this study achieved, results from 2010 were compared to the peak volume of outbreak-related news reports from HealthMap [13].

**Table 1:** Amazon Mechanical Turk Mumbai survey responses, July 16 - August 26 2010

Question & response categories	Results (N = 211), no (%)
1. Have you recently experienced any of the following symptoms?	

Fever	47 (22.3)

Chills	12 (5.7)

Sweats	5 (2.4)

Weakness	26 (12.3)

Severe fatigue	6 (2.8)

Enlarged spleen	2 (0.9)

Nausea	7 (3.3)

Vomiting	18 (8.5)

Dry cough	18 (8.5)

Aches	14 (6.6)

Muscle pain	19 ( 9.0)

Back pain	19 (9.0)

2. Approximately how many mosquito bites	Mean (95% CI)

have you had in the past 24 h?	14 (10.8-17.6)

3. Have you recently visited a doctor and/or were you recently diagnosed with malaria?	

Yes	78 (36.9)

No	128 (61.0)

No answer	5 (2.4)

4. If you answered YES in question 3, please indicate which type of malaria you had. If you don't know or are unsure, please skip this question.	

P. vivax	43 (20.4)

P. falciparum	18 (8.5)

5. Please check all that apply. Do you:	

Sleep under a bed net	15 (7.1)

Have standing pools of water near your home or place of work	4 (1.9)

Cover your skin when outside at dawn or dusk	0 (0.0)

Use mosquito repellent	12 (5.7)

Use a fan	29 (1.4)

Use a mosquito coil or other repellent for a room	15 (7.1)

6. Please enter your current location (City: Mumbai)	

Neighbourhood: (for example: Juhu, Worli, Santacruz, etc.)	(N = 205)

Juhu	17 (8.1)

Worli	11 (5.2)

Santacruz	10 (4.7)

Thane	2 (0.9)

Bandra	5 (2.4)

Andheri	6 (2.8)

Other/No answer	154 (75.1)

7. How old are you? Please enter your current age.	

< 20	89 (42.2)

20-24	94 (44.5)

25-29	17 (8.1)

30-34	10 (4.7)

35-40	0 (0.0)

> 40	1 (0.0)

No answer	1 (0.0)

## Results

**Figure 1:**
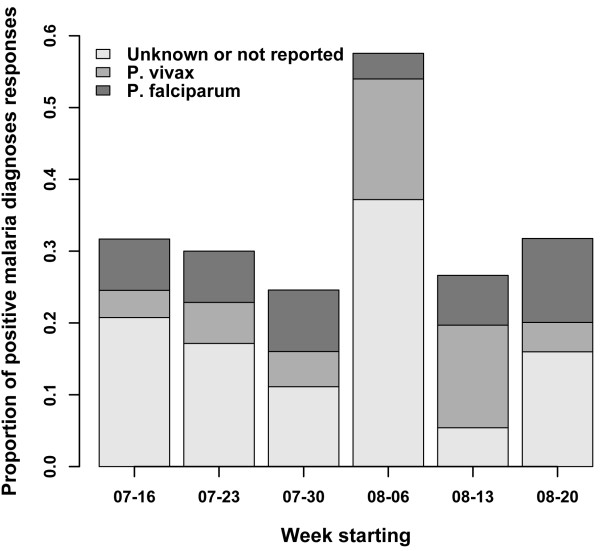
**Proportion of positive responses for malaria diagnoses by week from Amazon Mechanical Turk 2010 survey**. Aggregated responses by week and by proportion of Plasmodium types reported. AMT self-reported positive diagnoses peaked in the week starting August 6. The peak volume of related HealthMap reports occurred one week prior (week starting July 30). Proportion of Plasmodium reports from AMT also corresponded to available news and literature reports for the same time period

**Figure 2:**
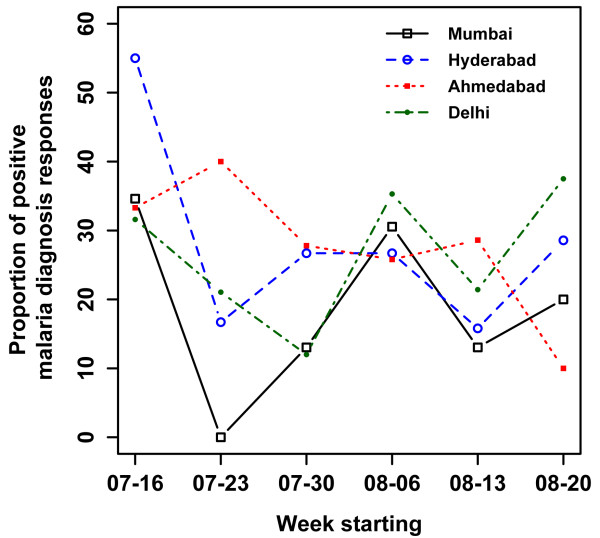
**Proportion of positive responses (%) for malaria diagnoses by week and city from Amazon Mechanical Turk 2011 survey**. In 2011 the study expanded to include responses from New Delhi, Ahmedabad, Hyderabad in addition to Mumbai. The responses from each city, showed no discernable peak for the same time period as the responses in 2010. 2011 cases in general were decreased from 2010

**Figure 3:**
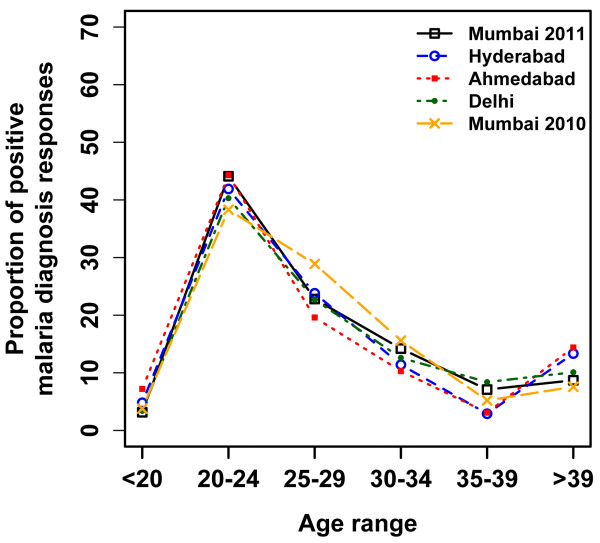
**Proportion of positive responses (%) for malaria diagnoses by age (2010 and 2011)**. For all cities and across both years of the study, the 20-24 age group had the highest proportion of positive malaria diagnosis responses. We are unaware of any other studies with as fine age resolution with which to compare the prevalence trends

**Figure 4:**
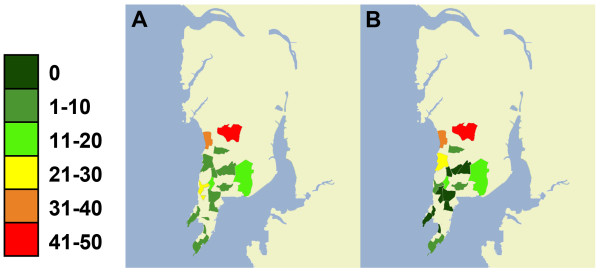
**Distribution of responses by neighborhood from 2010 and 2011 Mumbai surveys**. Responses from 2010 (A) illustrate all neighborhoods entered by all Turkers (in the city of Mumbai). In 2011 (B) the survey was altered to ask Turkers to select the neighborhood in which, or closest neighborhood to which they live

In 2010, 330 total responses were received from Turkers, and 442 in 2011 over the course of 42 days each year. Overall, 61.8% of the responses were from men and 37.1% from women. The youngest responder was 18 years, and eldest was 62 years. Responses were excluded from our results based the following criteria: repeat response (2010), Turker was located outside of the solicited area, or a response was given which contained contradictory information. Of the included responses, the proportion that reported positive malaria diagnosis peaked on the week starting August 6 in 2010 (Figure 1). The peak volume of related HealthMap reports occurred one week prior to the AMT peak of positive malaria diagnoses. Additionally, anecdotal evidence from media reports suggest that new cases dropped by 50% in the first two weeks of August compared to the end of July [14], aligning in time with the peak observed through AMT responses. There was no observable peak in the 2011 data (Figure 2) and news volume about Malaria in 2011 was much lower, attributed to the fact that there was no heightened outbreak that year. The distribution of *Plasmodium *species revealed through AMT self-report in 2010 was similar to that described in the literature on this region. Of the responses positive for malaria diagnosis from Mumbai, 59% (95% confidence interval (CI): 51.8-65.4) reported being diagnosed with *Plasmodium vivax *and 19% (95% CI: 14.0-25.0) with *Plasmodium falciparum*. The burden of *P.vivax *in India has been reported anywhere from 50%-69% of all malaria cases [15,16]. In the 2010 outbreak, 10-15% of cases in Mumbai were found to be *P. falciparum *[17]. Out of those who self-reported a positive malaria diagnosis, there were much fewer responses about *Plasmodium *species in 2011 (38.0% vs 78% in 2010). Overall prevalence in Mumbai went from 36.9% in 2010-22.0% in 2011 for the same time period, significantly decreasing (*p *= 0.006). Prevalence for age was highest in the 20-24 age category in 2010 and as well in all cities in 2011 (Figure 3). Similar overall prevalence trends were found in each in each city in 2011 (Table 2). In both years, it was possible to obtain prevalence information at the spatial level of the neighbourhood (Figure 4).

**Table 2:** Positive malaria diagnosis reports for Mumbai, New Delhi, Ahmedabad and Hyderabad (2011)

City	Overall prevalence (%)
Mumbai	22.0

New Delhi	26.0

Ahmedabad	25.5

Hyderabad	29.1

## Discussion

Numerous studies have examined how Internet searches can "predict the present", meaning that search volume correlates with contemporaneous events [18-20]. Specifically in the case of influenza, search volume was shown to estimate flu activity, which was not officially reported until two weeks later, and despite unknown flu status of the searchers. Building on this concept, a medium such as AMT allows for obtaining more detailed information beyond disease prevalence in real-time while harnessing the vast pervasiveness and convenience of the Internet. For instance, this study shows that AMT can be a way to garner public health information and at resolutions in time, space and demography that are unavailable in other forms of surveillance.

This work demonstrates the first use of harnessing micro-monetary incentives and online-reporting for public health surveillance. Traditionally AMT is used to recruit individuals to perform tasks difficult for artificial intelligence, such as in image and natural language processing. Previous studies investigating the efficacy of monetary incentives for performing tasks showed AMT provides a flexible and robust venue optimized for payment type [7]. The motivation for Turkers in India is more often monetary than for non-Indian Turkers (27% of Indians report requiring AMT income to make ends meet). Although financial motivation could lead to arbitrary responses, here we demonstrate that the data collected reflects other surveillance methods' findings for the outbreak period. Several studies have used AMT without a gold standard verification and have shown how to shape surveys, e.g. by including validation tests, to help ensure the quality of AMT responses [21]. Verifiable questions signal to users that their answers will be scrutinized, potentially both reducing invalid responses and increasing time-on-task. Experience from this study also suggests that public health surveillance via online self-reporting should also incorporate a structured set of verifiable questions to enable substantiation, particularly when other traditional surveillance methods may be deficient.

This study capitalizes on AMT's demographics; India is the second largest user base [22]. Additionally, Internet use and access, although increasing, has higher reach in urban areas. Further, AMT's user-base in India has an average age of 26-28 years, and Indian Turkers are substantially more likely to be male than US Turkers (two-thirds of Indian Turkers are male) [22].

The very small amounts of payment administered through AMT also have been shown to be sufficient to garner public health information from this population, thus demonstrating AMT as a way to perform a field study at a very reduced cost. The optimal amount of monetary incentives used to solicit public health information should be studied further, however, this kind of payment offers a drastically reduced cost for administering a field study over traditional methods [23]. In addition, this medium can easily and quickly reach remote subjects who may be underserved by traditional health infrastructure, where the majority of malaria deaths occur in India [5]. AMT is a particularly useful platform because it maintains anonymity of users, thereby assuring study subjects that sensitive personal health data will be kept private and secure.

Self-reporting is worth exploring due to likely differences in content and timing of self-reported versus physician-reported information [24]. Through self-reporting users gain more involvement with their own health, which can be important in fostering preventative health behaviours. Furthermore, self-report is facilitated by the rapid spread of consumer technology like mobile phones and eliminates delays by bypassing the chain-of-command relay structure of traditional public health surveillance [6].

Methods like AMT offer an epidemiologic tool with greatly reduced cost compared to traditional field surveys. Shown here, AMT can give unprecedented access to finely-resolved real-time public health information (daily, weekly) that would otherwise be unavailable and have vital implications for prevention and control measures. Taking advantage of a tool such as AMT for public health reporting on a particular environment and with a specific disease focus (here, malaria in India), can be useful as a complementary tool to existing and traditional public health infrastructure by providing focused outbreak investigation from particular groups. For malaria surveillance in particular, AMT could be used to investigate drug therapy adherence, which is a large issue in malaria relapse.

There is no available gold-standard with comparable temporal or spatial resolution which to confirm accuracy of the proportion of malaria infections, as garnered through AMT. HealthMap reports were one available source with similar resolution in time (daily). The outbreak peak, measured through volume of positive responses in AMT for 2010, was delayed compared to the volume of HealthMap reports. This could be due to the fact that there is more news reporting earlier in an epidemic period. In addition, by the time an outbreak peaks, awareness of the outbreak may then subsequently increase self-reporting response rate from the public.

In examination of the proportion of positive malaria diagnoses through AMT in 2010, the percentage of reported positive malaria diagnoses was markedly higher than the most relevant data (during the outbreak, from June 1-20, 8.4% of people from Mumbai examined tested positive, vs. 36.9% of AMT responses) [25]. The percentage of positive diagnoses from AMT in 2011 was significantly lower than in 2010. This corresponds to the trends conveyed by governmental organizations [14,26,27]; the number of cases dropped by 80.4% for the year until early August and the slide positivity rate, the measure of malaria incidence, dropped by 18.9%. Officially reported numbers of course only represent burden of the population using health care facilities. The lower proportion of the reports relaying the *Plasmodium *type in 2011 via AMT could be due to a slight change in wording of the survey from 2010-2011.

Media reports may underestimate disease prevalence, as some cases are not reported to a physician. Furthermore, some cases seen by physicians are not reported to regional offices [28]. Conversely, reports from AMT may also be biased due to a likely greater proportion of reports to physicians by the Turker population's demographics (age, education level, geographic concentration in urban areas and technology usage). The AMT responses may also be skewed by Turkers who have recently heard about malaria in the media and are more interested in a malaria-related HIT, or who might falsely believe that the researchers desire and better reward positive diagnosis reports.

In comparing spatial and demographics, no finely resolved official age prevalence information exists to compare our finding of the age-specific prevalence.

## Conclusions

This study provides a first look into using micro-financial incentives to promote public health reporting by the general public, with a focus on malaria in Mumbai, India. Due to the extremely small monetary values used as incentive payment and low overhead for the study, venues such as AMT provide a very cost effective method for running an epidemiological study at much lower expense than traditional field studies. Additionally, this study explores the use of an online medium through which to offer incentives. This type of medium is, and in the near future will become more, pervasive around the world. Online systems such as AMT and financial incentives may complement and even enhance traditional survey methods. Consequently the online medium is relevant both in communities with established surveillance systems as well as places where traditional surveillance infrastructure may be lacking.

The current demographics of AMT users make it particularly conducive for studying malaria in India. In addition, as with previous studies, this investigation finds that online reporting with small monetary incentives can be a successful medium for obtaining plentiful self-reported health information from individuals. Further exploration about incentives and their impact is imperative for building effective, real-time systems for gathering accurate information directly from the public.

## Competing interests

The authors declare that they have no competing interests.

## Authors' contributions

RC, VC, SRM, CCF and JSB conceived the study, VC, SB and RC implemented the study. RC and SB undertook data extraction and analysis, interpretation of results and drafted the manuscript. EC assisted in data analysis. SRM and JSB were involved in critical revision of the manuscript. All authors read and approved the final manuscript.

## Supplementary Material

Additional file 1 **Amazon Mechanical Turk Mumbai survey responses, July 16-August 26 2011**. Summarized results for each of the questions in which Turkers selected a response from a list of options Click here for file

Additional file 2 **Description of change from multiple batches of surveys (2010) to one release of the survey (2011)**. Click here for file
