# Supplementary material for: Online reporting for malaria surveillance using micro-monetary incentives, in urban India 2010-2011
Source: Malar J. 2012 Feb 13;11:43. doi: 10.1186/1475-2875-11-43 (PMC3305483; doi:10.1186/1475-2875-11-43)
Supplement: Additional file 1 — Amazon Mechanical Turk Mumbai survey responses, July 16-August 26 2011. Summarized results for each of the questions in which Turkers selected a response from a list of options [file 1475-2875-11-43-S1.DOCX]

Issue of duplicate responses in 2010: Between July 16 and August 26 2010, the Mumbai-specific HITs produced 330 responses, 64% of which were from unique users. Although AMT restricts Turkers to responding only once to an HIT, and because we offered multiple surveys in 2010 we could not ensure that the Turkers would respond to only one batch of the HITs on the same topic. However we found that typically, information reported in duplicate was consistent, corroborating accuracy of the reported information. We only made one deployment of surveys in 2011 to avoid the duplicate report issue.
