# Supplementary material for: Online reporting for malaria surveillance using micro-monetary incentives, in urban India 2010-2011
Source: Malar J. 2012 Feb 13;11:43. doi: 10.1186/1475-2875-11-43 (PMC3305483; doi:10.1186/1475-2875-11-43)
Supplement: Additional file 2 — Description of change from multiple batches of surveys (2010) to one release of the survey (2011). [file 1475-2875-11-43-S2.DOCX]

# Additional file 2 - Amazon Mechanical Turk Mumbai survey responses, July 16 – August 26 2011. Summarized results for each of the questions in which Turkers selected a response from a list of options.

| Question & response categories | **Mumbai results**  **(N = 127), no (%)** | | | | **New Delhi results**  **(N = 118), no (%)** | | | | | **Hyderabad results**  **(N = 103), no (%)** | | | **Ahmedabad results**  **(N = 94), no (%)** | | | |
| --- | --- | --- | --- | --- | --- | --- | --- | --- | --- | --- | --- | --- | --- | --- | --- | --- |
| 1.How old are you? | | | | | | | | | | | | | | | | |
| 2. Are you: | | | | | | | | | | | | | | | | |
| Male  Female | | | 81 (63.8)  46 (36.2) | | | 69 (58.5)  45 (38.1) | | | | 67 (65.0)  36 (35.0) | | | | | 56 (59.8)  37 (39.4) | |
| 3. Please enter the neighborhood of, or nearest to your residence (selection from drop-down list) | | | | | | | | | | | | | | | | |
|  | | Juhu  Worli  Santacruz  Colaba  Bandra  Andheri  Chembur  Borivali  Dadar  Thane | | 28 (22.0)  3 (2.4)  7 (5.5)  4 (3.1)  13 (10.2)  19 (15.0)  12 (9.4)  4 (3.1)  13 (10.2)  24 (18.9) | | Ashok Nagar  Alaknanda  Connaught Place  Khan Market  Pandav Nagar  Kabir Nagar  Vishwas Nagar  Civil Lines  Delhi Cantonment  Pitampura  Patel Nagar  Paschim Vihar  Model Town  RamKrishna Puram  Lajpat Nagar  Chandini Chowk | | 33 (28.0)  2 (1.7)  8 (6.8)  2 (1.7)  1 (0.8)  2 (1.7)  4 (3.4)  4 (3.4)  11 (9.3)  6 (5.1)  7 (5.9)  2 (1.7)  5 (4.2)  6 (5.1)  16 (13.6)  9 (7.6) | | Jubilee Hills  Banjara Hills  Indira Park  Sanathnagar  Saidabad  Kurmaguda  Amberpet  Bholakpur  Uppuguda  Puranapul  Jiyaguda | 29 (28.2)  10 (9.7)  21 (20.4)  10 (9.7)  11 (10.7)  4 (3.9)  8 (7.8)  3 (2.9)  4 (3.9)  1 (1.0)  2 (1.9) | | | Naroda  Bhandra  Asarwa  Mahadev Nagar  Ranip  Vastrapur  Memnagar  Paldi  Vejalpur  Isanpura  Thakkarbapa Nagar  Dakshini Society  Ambavadi  Nicol  Sherkotda  Chanakyapuri | | 7 (7.4)  8 (8.5)  32 (34.0)  4 (4.3)  4 (4.3)  5 (5.3)  4 (4.3)  6 (6.4)  2 (2.1)  2 (2.1)  2 (2.1)  4 (4.3)  9 (9.6)  1 (1.1)  0 (0.0)  4 (4.3) |
| 4. In the past 30 days, have you experienced any fever? | | | | | | | | | | | | | | | | |
| Yes  No | 47 (37.0)  27 (21.3) | | | | 49 (41.5)  67 (56.8) | | | | | 41 (39.8)  10 (9.7) | | | 31 (33.0)  61 (64.9) | | | |
| 5. In the past 30 days, were you officially diagnosed with malaria (at a hospital, by a healthcare worker, by a doctor)? | | | | | | | | | | | | | | | | |
| Yes  No | 28 (22.0)  99 (78.0) | | | | 31 (26.3)  84 (71.2) | | | | | 30 (29.1)  74 (71.8) | | | 24 (25.5)  67 (71.3) | | | |
| 6. If you were officially diagnosed with malaria in the last 30 days, indicate on which date you were diagnosed. If you don’t remember, proceed to the next question. | | | | | | | | | | | | | | | | |
| 7. If you had malaria in the past 30 days, which type of malaria did you have? If you don’t know, don’t remember, or weren’t told please select unknown. | | | | | | | | | | | | | | | | |
| P. vivax  P. falciparum | 5 (3.9)  7 (5.5) | | | | 3 (2.5)  5 (4.2) | | | | | 8 (7.8)  7 (6.8) | | | 3 (3.2)  4 (4.2) | | | |
| 8. In the past 30 days, was anyone besides you in your home diagnosed with malaria? | | | | | | | | | | | | | | | | |
| Yes  No | 35 (27.5)  87 (68.5) | | | | 32 (27.1)  80 (67.8) | | | | | 25 (24.3)  71 (68.9) | | | 22 (23.4)  71 (75.5) | | | |
| 9. How is malaria spread? (pick one) | | | | | | | | | | | | | | | | |
| Person-to-person contact  Airborne  Via mosquitoes  Via animals | 8 (6.3)  5 (3.9)  108 (85.0)  0 (0.0) | | | | 6 (5.1)  3 (2.5)  104 (88.1)  1 (0.8) | | | | 7 (6.8)  3 (2.9)  88 (85.4)  3 (2.9) | | | | 10 (10.6)  1 (1.1)  79 (84.0)  3 (3.2) | | | |
| 10. How have you heard of malaria prevention measures? Pick all that apply. | | | | | | | | | | | | | | | | |
| I have not heard of preventative measures  From government awareness programs  From medical or other health workers  From education/school programs  From the news or other media  Other | | | 22 (17.3)  64 (50.4)  52 (40.1)  52 (40.9)  67 (52.8) | | | | 14 (11.8)  60 (50.8)  46 (39.0)  49 (41.5)  50 (42.4) | | | 15 (14.6)  56 (54.4)  45 (43.7)  57 (55.3)  47 (45.6) | | 15 (16.0)  51 (54.3)  52 (55.3)  50 (53.2)  46 (48.9) | | | | |
